# Impaired AQP2 trafficking in *Fxyd1* knockout mice: A role for FXYD1 in regulated vesicular transport

**DOI:** 10.1371/journal.pone.0188006

**Published:** 2017-11-20

**Authors:** Elena Arystarkhova, Richard Bouley, Yi Bessie Liu, Kathleen J. Sweadner

**Affiliations:** 1 Laboratory of Membrane Biology, Dept. of Neurosurgery, Massachusetts General Hospital and Harvard Medical School, Boston, Massachusetts, Unites States of America; 2 Center for Systems Biology, Program in Membrane Biology and Division of Nephrology, Massachusetts General Hospital and Harvard Medical School, Boston, Massachusetts, United States of America; University of Utah School of Medicine, UNITED STATES

## Abstract

The final adjustment of urine volume occurs in the inner medullary collecting duct (IMCD), chiefly mediated by the water channel aquaporin 2 (AQP2). With vasopressin stimulation, AQP2 accumulation in the apical plasma membrane of principal cells allows water reabsorption from the lumen. We report that FXYD1 (phospholemman), better known as a regulator of Na,K-ATPase, has a role in AQP2 trafficking. Daytime urine of *Fxyd1* knockout mice was more dilute than WT despite similar serum vasopressin, but both genotypes could concentrate urine during water deprivation. FXYD1 was found in IMCD. In WT mice, phosphorylated FXYD1 was detected intracellularly, and vasopressin induced its dephosphorylation. We tested the hypothesis that the dilute urine in knockouts was caused by alteration of AQP2 trafficking. In WT mice at baseline, FXYD1 and AQP2 were not strongly co-localized, but elevation of vasopressin produced translocation of both FXYD1 and AQP2 to the apical plasma membrane. In kidney slices, baseline AQP2 distribution was more scattered in the *Fxyd1* knockout than in WT. Apical recruitment of AQP2 occurred in vasopressin-treated *Fxyd1* knockout slices, but upon vasopressin washout, there was more rapid reversal of apical AQP2 localization and more heterogeneous cytoplasmic distribution of AQP2. Notably, in sucrose gradients, AQP2 was present in a detergent-resistant membrane domain that had lower sedimentation density in the knockout than in WT, and vasopressin treatment normalized its density. We propose that FXYD1 plays a role in regulating AQP2 retention in apical membrane, and that this involves transfers between raft-like membrane domains in endosomes and plasma membranes.

## Introduction

Maintenance of salt and water balance is an essential physiological function of the kidney. While regulation of Na^+^ homeostasis requires the orchestrated work of multiple Na^+^ transporters along the nephron, water reabsorption across the tubular epithelium is almost entirely dependent on the activity of water channels, aquaporins (AQPs). Five AQPs have been identified in kidney (reviewed in [1]). At baseline, about 90% of the filtered water is passively reabsorbed in proximal tubules and in the thin descending limb of Henle via AQP1 channels. Adaptive control of urine composition, however, occurs in collecting duct (CD), and is under the control of AQP2 (reviewed in [2,3]).

Both expression and localization of AQP2 in principal cells of collecting duct are regulated by vasopressin (VP, anti-diuretic hormone), although other stimuli are also implicated (angiotensin II, prostaglandins, dopamine, oxytocin, atrial natriuretic peptide). It is understood that “baseline” is a physiological state in which vasopressin levels and AQP2 trafficking are subject to frequent minor adjustments [3]. Final water reabsorption from the CD requires AQP2 to be at the apical plasma membrane. This is a transient event, though, comprising the regulated trafficking of AQP2-bearing vesicles to the apical membrane, docking and fusion of vesicles with the apical membrane (exocytosis), and regulated AQP2 retrieval (endocytosis) [4,5]. Major defects in AQP2 biosynthesis or targeting can lead to serious clinical conditions, such as significant water loss in nephrogenic diabetes insipidus or excessive water retention in the syndrome of inappropriate antidiuretic hormone secretion (reviewed in [6]).

In addition to the aquaporins, there are other protein families whose members show regionally distinct patterns of expression along the renal tubule and may, therefore, be involved in regulating functionally important cellular activities in a segment-specific manner. One striking example is the FXYD protein family, which were shown to associate with and regulate the Na,K-ATPase [7,8]. Most abundant are FXYD2 (proximal tubule, medullary thick ascending limb, and distal convoluted tubule) and FXYD4 (collecting duct), but FXYD1 and FXYD5 have also been detected [9–12]. In rodent kidney, FXYD1 was identified in the juxtaglomerular apparatus in afferent arterioles and lacis cells, which are involved in regulation of glomerular filtration rate and blood pressure [11]. FXYD1 was also detected in rat IMCD cells by LC-MS in a large-scale proteomic analysis [13].

FXYD1 (phospholemman, PLM) is a small single-span membrane protein originally discovered in sarcolemma of cardiac and skeletal muscle as a target of multiple protein kinases [14–16]. It is also expressed in smooth muscle, liver, lung, brain, adipocytes, and a secretory epithelium, choroid plexus (reviewed in [8]). FXYD1 is the only member of the family known to possess multiple phosphorylation sites that, individually or in combination, can be phosphorylated by kinases [17,18] and dephosphorylated by phosphatases [19,20]. Non-phosphorylated FXYD1 reduces Na,K-ATPase by reduction of Na^+^ affinity, the rate limiting factor for activity, and its phosphorylation increases Na,K-ATPase activity, modulating the baseline activity to fine-tune the Na,K-ATPase in response to physiological need [21–23]. FXYD1 can also be palmitoylated [24,25]. Phosphorylation, palmitoylation, and oxidative glutathionylation of FXYD1 are interacting, sometimes competing, modifications of FXYD1, consistent with its role as a target of converging signals [24,26,27].

In the heart, FXYD1 regulates not only Na,K-ATPase, but also sodium-calcium exchanger and L-type calcium channels [28–30], and it exists also in a separate pool [31]. Adult mice with global knockout of the *Fxyd1* gene exhibit mild cardiac hypertrophy characterized by increased cardiac mass and mildly depressed contractile function, but normal blood pressure [32,33]. Here we describe an unexpected renal phenotype of the *Fxyd1*^-/-^ mice: deficient daytime concentration of urine. We present an initial mechanistic definition of the physiological phenomenon by testing a series of basic hypotheses. A role for FXYD1 in AQP2 trafficking is suggested by their co-recruitment to the apical plasma membrane in response to vasopressin and by more rapid reversal in the *Fxyd1* knockout.

## Materials and methods

### Animals and procedures

All mouse protocols were approved by the Massachusetts General Hospital Subcommittee on Research Animal Care and were in accordance with the National Institutes of Health's *Guide for the Care and Use of Laboratory Animals*. *Fxyd1*^*-/-*^ mice were generated by targeted replacement of exons 3–5 [32]. They were used from the 8^th^ to the 10^th^ backcross to the C57BL/6NCrl mouse strain (Charles River Laboratories, Wilmington, MA). Homozygous mutants, littermate controls, and controls from other litters in the same cohort were produced from heterozygote parents that in turn were from back-crosses to wild types from the supplier. Offspring were genotyped by PCR amplification of ear punch DNA taken at weaning. Mice were given regular diet (0.3% Na^+^; ProLab IsoPro RMH 3000), had free access to water, and were on a 12 hour light/dark cycle, lights on from 7 am to 7 pm. Breeding and longevity were unaffected in the knockout.

Water deprivation was for 36 hours with access to solid food for individually-housed mice with fresh litter. Injection with the vasopressin type 2 receptor (V2R)-selective agonist dDAVP [deamino-Cys^1^,D-Arg^8^-vasopressin (Sigma Aldrich, St. Louis, MO)], 1 μg/kg, was intraperitoneal. Vasopressin in blood samples was determined with an Arg^8^-vasopressin ELISA Kit (Assay Designs, Enzo Life Sciences, Farmingdale, NY). Urine osmolality was measured in duplicate using a Vapro 5520 vapor pressure osmometer (Wescor, Logan, UT).

### Antibodies

Rabbit antiserum K1 was used to detect α1 subunits of Na,K-ATPase on blots, as validated elsewhere [34]. Mouse monoclonal antibody 9A7 was used for immunofluorescence detection of α1 (gift of Maureen W. McEnery, Case Western Reserve University, Cleveland, OH) (specificity documented in [35]). Rabbit antibody PLM-C2 was raised against a C-terminal peptide that includes the phosphorylation sites (gift of Dr. Joseph Cheung, Temple University Lewis Katz School of Medicine, Philadelphia, PA), and was used to detect non-phosphorylated FXYD1 on blots. Its characterization and phospho-sensitivity has been published [36–38]. Rabbit antibody PLM-C1 was raised against the same peptide (gift of Dr. Larry Jones, Krannert Institute of Cardiology, Indiana University, Indianapolis, IN) [39]. It was utilized to detect non-phosphorylated FXYD1 on sections. Its specificity was defined in [39] and its phospho-sensitivity and lack of reactivity in the knockout is documented on blots in Figs A and B in S1 File. Although C1 and C2 antibodies are very similar, the affinity-purified C1 gave a stronger signal in immunofluorescence. Because C1 was in limited supply, the C2 antibody was used on blots. FXYD1 phosphorylated at Ser63 and Ser68/Thr69 were recognized with sheep phospho-specific antibodies CP63 and CP689, respectively (gift of Dr. Will Fuller, Medical Research Institute, University of Dundee, UK), which were characterized previously [18]; blots negative on knockout tissue are shown in Fig C in S1 File. Goat antibodies against AQP2 were from Santa Cruz Biotechnology (sc-9882, Santa Cruz, CA). This antibody was validated using AQP2 transfected and non-transfected cells [40].

### Immunofluorescence

Cryostat sections (5 μm thickness) of paraformaldehyde-lysine-periodate (PLP)-fixed kidneys and slices were treated with 1% SDS in phosphate saline buffer for antigen retrieval [41], and then dual stained with rabbit antibody PLM-C1 and mouse anti-Na,K-ATPase α1; or PLM-C1 and goat antibody anti-AQP2. Detection was with Alexa Fluor-conjugated secondary antibodies (Jackson ImmunoResearch, West Grove, PA or Life Technologies, Grand Island, NY). Tissue slices were mounted with Vectashield containing Dapi (Vector Laboratories, Burlingame, CA), or TO-PRO-3 (ThermoFisher) to counter-stain nuclei. Images were collected on a Zeiss LSM Pascal 5 or a Nikon A1R Resonant Scanning confocal system.

### Sample preparation and gel analysis

All urine, blood, and tissue samples were collected between 12 noon and 4 pm (ZT5-9) except kidneys collected 16 hours after vasopressin injection. Crude membrane preparations were obtained from inner medulla by homogenization in SET buffer containing 250 mM sucrose, 1 mM EDTA and 10 mM Tris-HCl, pH 7.4, and differential centrifugation at 3,000 x g, 15 min, at 4°C (Sorvall, SS-34), followed by centrifugation of the supernatant at 100,000 x g, 30 min, at 4°C (Beckman, Ti 70.1). All buffers contained complete protease inhibitor cocktail (Roche Diagnostics, Indianapolis, IN) and protein phosphatase inhibitor cocktail I (Sigma Aldrich, St. Louis, MS). Final pellets were resuspended in SET. Proteins were resolved on 4–12% NuPage MES SDS gels (Life Technologies, Grand Island, NY), transferred to nitrocellulose, and incubated with specific antibodies. Detection was with chemiluminescence using a digital imaging system, ImageQuant LAS4000 (GE Healthcare Biosciences, Pittsburgh, PA). Quantification was with ImageQuant TL image analysis software. Molecular weight markers were SeeBlue Plus2 (ThermoFisher Scientific).

Preparation of lysates was as previously described [13]. Inner medulla from freshly isolated kidneys was homogenized in a buffer containing 320 mM sucrose, 10 mM HEPES, pH 7.5, 1 mM EGTA, 1 mM EDTA and complete protease inhibitor cocktail followed by centrifugation at 3,000 x g at 4°C for 10 minutes. Triton X-100 and dithiothreitol (DTT) were added to supernatants to a final concentration 1% and 1 mM, respectively. Cell lysates were the supernatants of a final centrifugation at 16,000 x g for 30 min at 4°C.

DRM, detergent-resistant membranes, were prepared by nonionic detergent extraction followed by discontinuous sucrose gradient centrifugation [13]. The procedures were carried out at 4°C in TNE buffer containing 25 mM Tris-HCl, pH 7.4, 150 mM NaCl, and 5 mM EDTA and protease inhibitor cocktail. Inner medullas from freshly isolated mouse kidney were homogenized in SET buffer. Nuclei and unbroken cells were removed by centrifugation at 1,000 x g for 15 min, and supernatant was collected after centrifugation at 200,000 x g for 30 min, 4°C. The pellet was solubilized in 0.5 ml TNE buffer containing 1% Triton X-100 for 30 min at 4°C. Solubilized material (DRM-enriched membranes) was passed through a 22 gauge needle and combined with an equal volume of TNE buffer containing 80% sucrose (w/v) to make a final 40% sucrose solution, which was placed at the bottom of a centrifuge tube, overlaid with 3 ml of 30% sucrose in TNE buffer followed by 1 ml of 5% sucrose in TNE. Centrifugation was at 200,000 x g overnight at 4°C (swinging bucket rotor SW 50.1, Beckman Coulter, Fullerton, CA). Fractions (0.5 ml each) were collected from the top the gradient and equal volumes were resolved by gel electrophoresis and analyzed on immunoblots.

### Dephosphorylation analysis *in situ*

Fresh-frozen cryosections (10 μm) from wild type or *Fxyd1*^-/-^ kidneys were incubated with 100 μl lambda-phosphatase reaction buffer (50 mM Tris-HCl, pH 7.6, 100 mM NaCl, 0.1 mM EGTA, 2 mM dithiothreitol and 0.01% Brij 35) for 5 min at room temperature [42]. The incubation solution was changed to a dephosphorylation solution (100 μl) containing 400 U of lambda protein phosphatase (λPP) (New England Biolabs, Ipswich, MA), 2 mM MnCl_2_, lambda-protein phosphatase reaction buffer, and a protease inhibitor cocktail (Sigma-Aldrich, St. Louis, MO). The slides were incubated for 1 hour at 30°C, followed by rinse with 50 mM Tris-HCl, pH 7.5 and 0.1 mM EDTA, and fixed with 2% PLP. Slides processed the same way without phosphatase were used as controls.

### *In situ* kidney slice preparation and treatment

Wild type or *Fxyd1*^-/-^ mice were anesthetized with sodium pentobarbital (50 mg/kg i.p.). The blood was washed out by intraventricular perfusion using Hanks’ balanced salt solution (HBSS), pH 7.4, at 37°C, equilibrated with 5% CO_2_/95% O_2_. Transverse kidney slices were cut using a Stadie-Riggs slicer (Thomas Scientific, Swedesboro, NJ) [43]. Slices (except those studied at baseline) were equilibrated in HBSS for 15 min at 37°C to allow washout of residual endogenous vasopressin and return to a minimally-stimulated state before transfer to a fresh vial containing the V2R-selective agonist dDAVP [deamino-Cys^1^,D-Arg^8^-vasopressin (10^−9^ M)]. 15 min later, 3 slices were removed and fixed by immersion in PLP overnight at 4°C. The rest of the slices were transferred to a fresh vial containing HBSS for washout at 37°C (15 min, 30 min, 60 min, and 120 min) followed by PLP fixation as above. The fixed slices were rinsed several times in phosphate buffered saline, pH 7.4, and stored in the same buffer with 0.02% sodium azide at 4°C before processing by sectioning and immunostaining.

### Statistical analysis

Results were analyzed with unpaired Student’s *t*-test and were expressed as means of 4–6 independent experiments ± SEM. A two-tailed *P* value < 0.05 was considered significant.

## Results

### Renal phenotype of *Fxyd1*^*-/-*^ mice

**Fig 1:**
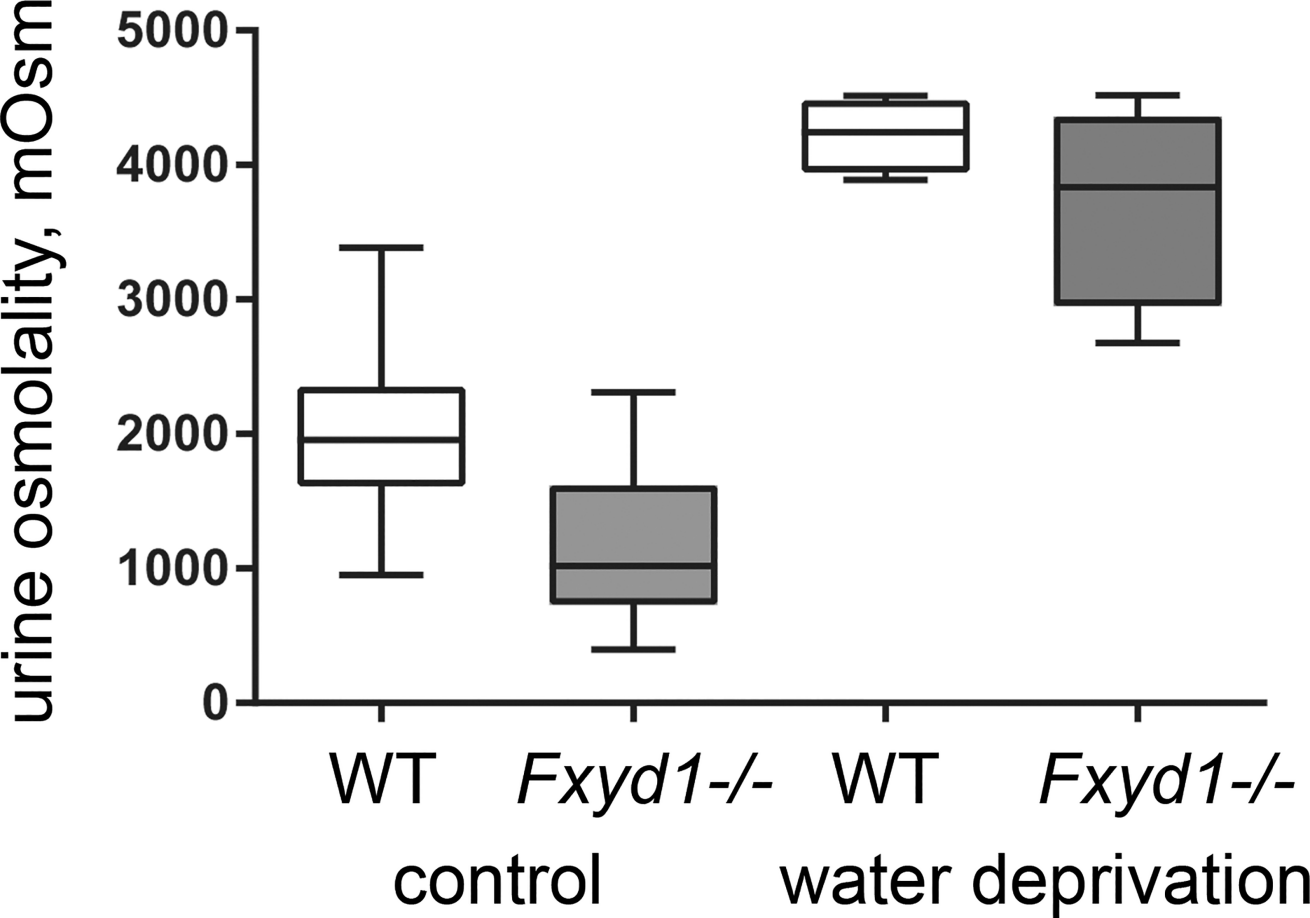
FXYD1 is implicated in urinary concentration. Box and whisker plot of the osmolality of daytime (afternoon) samples of male mouse WT and knockout (KO) urine at baseline and after water deprivation, showing a difference in baseline osmolality but the near-normal ability of the KO to concentrate its urine. The asterisks indicate a P value of <0.0001, 2-tailed Student’s *t*-test. When calculated as average ± SEM the results were as follows. WT control conditions, 2,019 ± 143, n = 20; KO control conditions 1,161 ± 123, n = 21; Student’s *t*-test, P < 0.0001. WT after 36 hours water deprivation, 4,224 ± 140, n = 8; KO water deprivation, 3,716 ± 383, n = 8; *t*-test, P = 0.26. Female mice were also tested in control conditions, and the results for baseline osmolality were WT, 2,169+/-92, n = 7; KO, 1,085+/-108, n = 7, *t*-test P < 0.001.

As reported earlier, mice with global deletion of the FXYD1 subunit of Na,K-ATPase are viable and fertile [32]. We confirmed that *Fxyd1*^*-/-*^ mice (now on the C57Bl/6NCrl strain background) did not show significant elevation in blood pressure compared to WT at baseline: 86.1 ± 1.0 vs. 89.5 ± 0.9 mm Hg (males, n = 8 per group) and 97.7 ± 2.3 vs. 99.0 ± 1.9 (females, n = 7 per group). Spot measurements of urine samples in the afternoon (ZT6-8), however, revealed that under basal conditions, and unlike the wild type, *Fxyd1*^*-/-*^ mice produced dilute urine. The daytime (sleep period) difference in urine osmolality was highly significant (Fig 1 shows medians, quartiles, and range, and means ± SEM are given in the legend). The genetic difference was present in both males and females. Urine concentration is primarily regulated by vasopressin produced in the hypothalamus and released from the posterior pituitary.

The first hypothesis tested was that there might be a reduction of circulating vasopressin in the *Fxyd1* knockout. There appear to be some FXYD1-stained cells in the supraoptic nucleus, which provides vasopressin to the posterior pituitary (Allen Brain Atlas, P56 mouse brain, Fxyd1-68632918, http://mouse.brain-map.org/). Measurement of baseline circulating vasopressin levels in plasma did not, however, reveal a statistically significant difference between wild type and knockout mice (males): 73.6 ± 10.3 pg/ml (n = 10) vs. 77.5 ± 5.0 pg/ml (n = 9), respectively. Vasopressin release is increased physiologically in response to an increase in plasma osmolality or a reduction in blood volume, so we further tested the hypothesis that *Fxyd1*^*-/-*^ mice may have a defect in vasopressin biosynthesis or release by subjecting animals to water deprivation. As shown in Fig 1, after 36 hours the osmolality of urine from *Fxyd1*^*-/-*^ mice was elevated to a level close to that of wild type littermates, although variability was larger. This is consistent with a near-normal vasopressin response, and with the mild phenotype.

**Fig 2:**
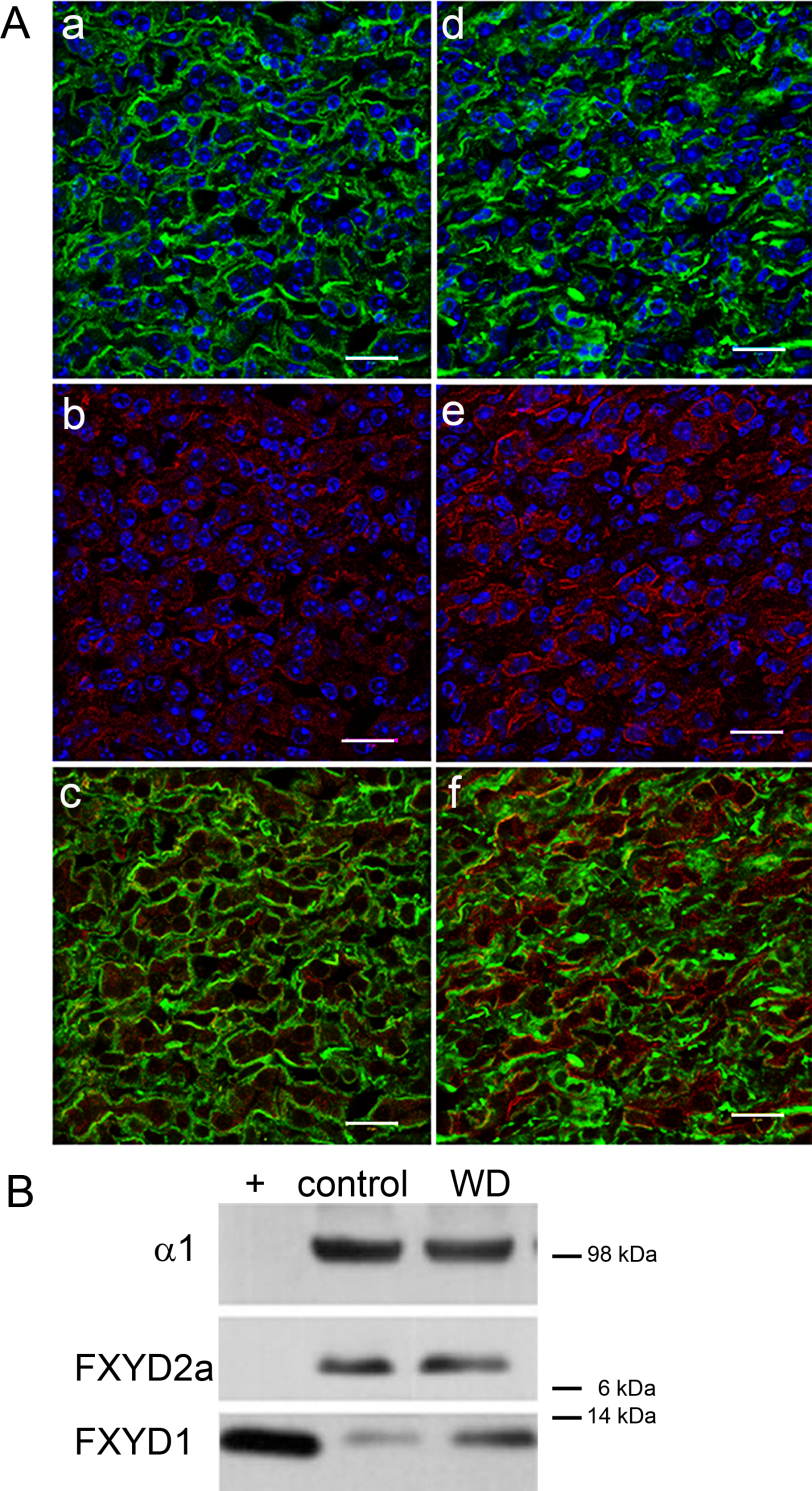
Wild type mice were deprived of water for 36 hours to elicit water conservation mechanisms. (A) Water deprivation revealed an intracellular pool of FXYD1 that was mostly not co-localized with basolateral Na,K-ATPase in mouse IMCD. Kidney sections from male WT control (a-c) or water deprived mice (d-f) were stained for α1 subunit of Na, K-ATPase (a,d) and FXYD1 (b,e). FXYD1 detection with the PLM-C1 antibody was enhanced in water-deprived mice. Nuclei were co-stained with TO-PRO-3 (blue). Bars, 20 microns. The images were enhanced and sharpened by subjecting the entire field to the high-pass filter in Adobe Photoshop. (B) Membranes from inner medulla from control or water-deprived (WD) animals were tested on blots with antibodies specific to the α1 subunit of Na,K-ATPase, FXYD2a, or FXYD1 (PLM-C2). (+) Canine cardiac sarcolemma was used as a positive blot control for FXYD1. The blot is representative of three independent experiments.

Previously FXYD1 was detected by immunofluorescence only in the juxtaglomerular apparatus in rats [11]. We next tested the hypothesis that for an effect on urine concentration, FXYD1 should be detected in inner medullary collecting duct of WT mice. Fig 2A shows double labeling of inner medulla from control (a-c) and water deprived (d-f) WT mice with anti-Na,K-ATPase α1 (a,d) and anti-FXYD1 (b,e). In WT, staining for FXYD1 was light (b), and a surprisingly large fraction of FXYD1 was not co-localized with basolateral Na,K-ATPase (c). Water-deprived mice showed no change in the distribution or intensity of Na,K-ATPase α1 labeling (d), but there was a striking increase in signal for FXYD1, some of which appeared to be in the apical membrane as well as the cytoplasm and basolateral membrane (f). The separation of FXYD1 from Na,K-ATPase before and during the response suggested that modulation of Na,K-ATPase activity is not implicated in the knockout’s phenotype.

Western blot analysis of kidney inner medulla from water-deprived wild type mice also revealed an enhanced signal for FXYD1 protein compared to WT controls (2.9 ± 0.6 fold increase, n = 3), while no difference was seen in the α1 or FXYD2a subunits of Na,K-ATPase that are abundant in kidney (Fig 2B). The enhanced FXYD1 signal in water deprivation was thus detected by two methods and two different antibodies. The increase in FXYD1 signal on sections and blots in Fig 2 could suggest an increase in FXYD1 protein expression due to water deprivation, but as will be seen, further experiments indicated that it was instead due to unmasking of epitopes blocked by phosphorylation.

### FXYD1 in IMCD was in a partially phosphorylated state

**Fig 3:**
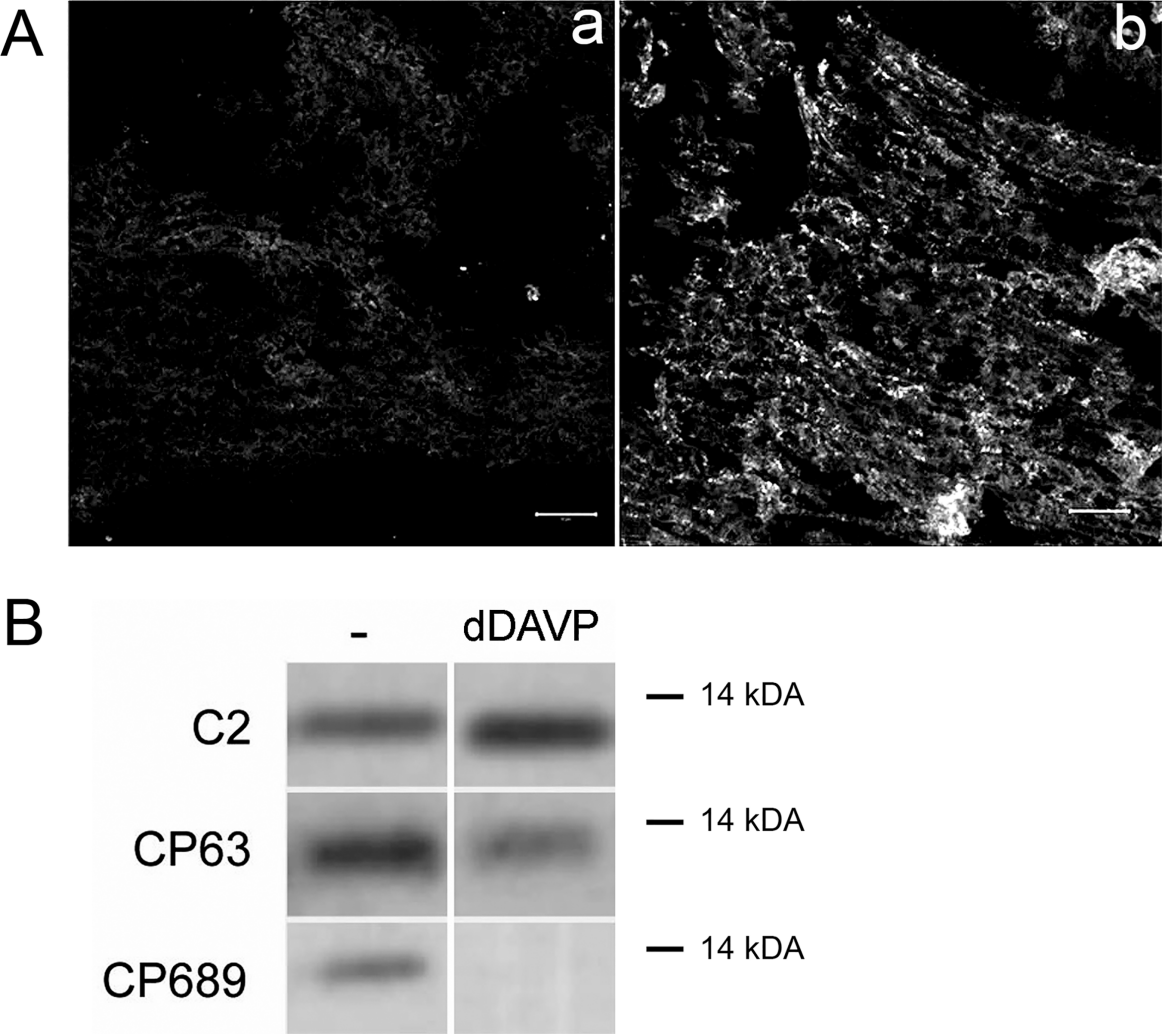
In wild type IMCD, FXYD1 was phosphorylated at the basal state and dephosphorylated by vasopressin treatment. (A) Dephosphorylation of FXYD1 by exogenous phosphatase *in situ* revealed enhancement of detection by PLM-C1 antibody, which is specific for dephospho-FXYD1. Fresh-frozen kidney sections from male wild type mice were treated with vehicle (a) or Lambda protein phosphatase (b), fixed with 4% PLP, and stained with PLM-C1. The data confirm that FXYD1 is phosphorylated at basal conditions. Bars, 50 microns. (B) FXYD1 phosphorylation under basal conditions was detected on blots. Wild type mice were injected with either vehicle (-) or dDAVP (IP, 1 μg/kg). Four hours post-injection membranes from inner medulla were prepared and tested on Western blots with PLM-C2 antibody, also specific for non-phosphorylated protein, and FXYD1 phospho-specific antibodies CP63 and CP689 that recognize phosphorylated Ser63 and Ser68/Thr69 [18], respectively. Vasopressin elicited reciprocal changes in antibody signal.

If FXYD1 has a role in IMCD downstream of vasopressin receptor (V2R) activation, it is reasonable to hypothesize that a change in FXYD1 phosphorylation could be detected. This was tested with a set of anti-FXYD1 antibodies that either require phosphorylation or are blocked by it. FXYD1 is a target of multiple protein kinases [17]. PLM-C1 and PLM-C2 antibodies were raised against the same peptide, GTFRSSIRRLSTRRR [36,39], which contains the serine and threonine phosphorylation sites. Bulky and negatively-charged phosphate groups greatly affect antibody recognition. Phosphorylation largely blocks the binding of both antibodies (Fig A in S1 File for PLM-C1; ref. [38] for PLM-C2), and an increase in signal could, therefore, be due to a reduction in phosphorylation. Fig 3A reveals the high baseline phosphorylation status of FXYD1 *in situ* in IMCD. Fresh-frozen kidney sections from WT mice were incubated without (a) and with (b) lambda protein phosphatase to dephosphorylate proteins immediately after sectioning, then were post-fixed and stained. The *in situ* dephosphorylation increased the detection of FXYD1 in inner medulla with PLM-C1 antibody (Fig 3A). In a complementary approach, antibodies specific for dephosphorylated and phosphorylated forms of FXYD1 were used on blots of inner medulla samples from wild type mice with and without dDAVP treatment (Fig 3B). FXYD1 was phosphorylated under basal conditions as indicated by staining with phosphospecific antibodies CP63 and CP689, which recognize sites phosphorylated by PKC and several other kinases. In samples from mice 4 hours after injection of dDAVP, reaction with the CP689 antibody was abolished and reaction with CP63 antibody was reduced, and there was a complementary increase in the binding of PLM-C2 antibody. The data show that FXYD1 is expressed in inner medulla, and that under basal conditions there is already some phosphorylation at multiple sites. Water deprivation or treatment with dDAVP stimulated dephosphorylation of FXYD1. Reduction in the phosphorylation level detected by CP63 and CP689, together with an increase in the signal for PLM-C2 antibody, indicated that basal phosphorylation of FXYD1 is a target for vasopressin-induced protein phosphatase activity. Stimulation of both kinases and phosphatases by vasopressin in the kidney is well established, as discussed below.

### AQP2 in *Fxyd1*^*-/-*^ mice

Upon release into the bloodstream, vasopressin binds to V2R receptors in basolateral membranes of renal collecting duct principal cells. In the classic model, the subsequent increase in the level of cAMP activates protein kinase A, activating a pathway that results in accumulation of the water channel aquaporin 2 (AQP2) at the apical membrane [44–46]. This process is necessary for increasing apical membrane water permeability, which is required for concentrating the urine. Our next hypothesis was that deletion of FXYD1 interfered with normal vasopressin-regulated trafficking of AQP2 in collecting ducts, and this could be responsible for the production of dilute urine in the knockout mouse. To resolve steps in AQP2 trafficking, we studied responses as a function of time.

**Fig 4:**
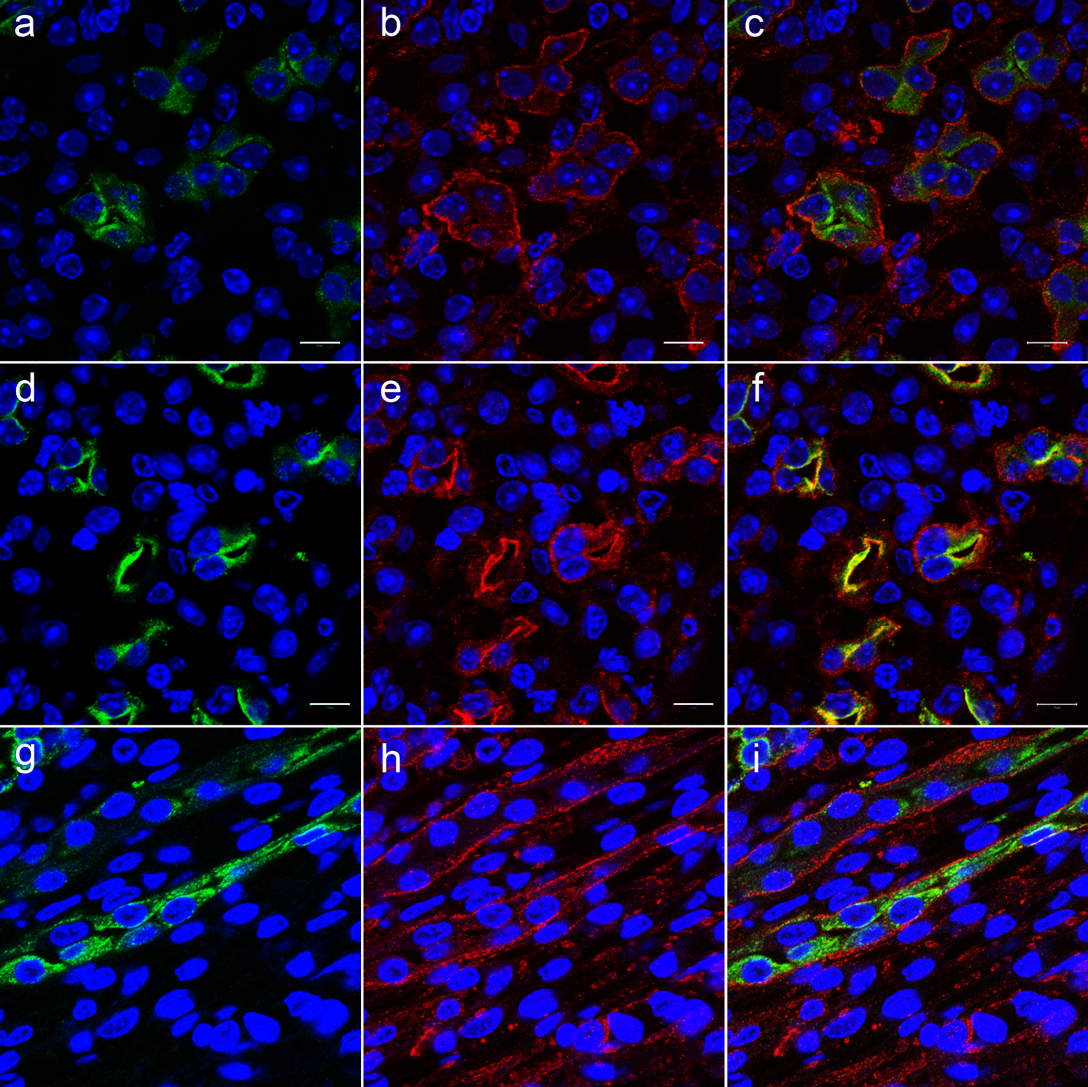
Vasopressin induced trafficking of FXYD1 in wild type IMCD *in vivo*. Wild type mice were injected with dDAVP (1 μg/kg) and sacrificed at 0 time (a-c), 4 hours (d-f), and 16 hours (g-i) post-injection. Fixed kidneys were sectioned, and slides were stained with antibodies against AQP2 (a,d,g) and PLM-C1 antibodies against FXYD1 (b,e,h). dDAVP stimulated the trafficking of FXYD1 from an intracellular location towards apical membrane. Co-localization of FXYD1 and AQP2 at apical membrane is seen in yellow after 4 hours (f). This figure is representative of 3 independent experiments. Nuclei were labeled with TO-PRO-3. Bars, 10 μm.

*In vivo*, colocalization of FXYD1 and AQP2 in IMCD was probed in sections of kidney from WT mice at baseline, 4 hours, and 16 hours after intraperitoneal injection of dDAVP. Fig 4 shows double staining for AQP2 (a,d,g,) and FXYD1 (b,e,h,) and merged images (c,f,i). Under basal conditions (c), there was a clear spatial separation: while the stain for FXYD1 was mostly localized in proximity to basolateral membranes with some protein retained intracellularly, AQP2 was seen mostly in intracellular compartments closer to the apical pole of the cells. Stimulation with dDAVP resulted in substantial rearrangement of both proteins within IMCD cells. The expected accumulation of AQP2 in the apical membrane was seen at 4 hours post-injection (f). Remarkably and unexpectedly, a large fraction of FXYD1 stain was likewise apical. Some intracellular and basolateral staining remained. The yellow in (f) is co-localization of the proteins at apical membrane. Fig 4G–4I demonstrates that 16 hours post-injection a reverse trafficking of both proteins had occurred in WT mice: most FXYD1 was detected near the basolateral membrane, and AQP2 was again generally intracellular.

**Fig 5:**
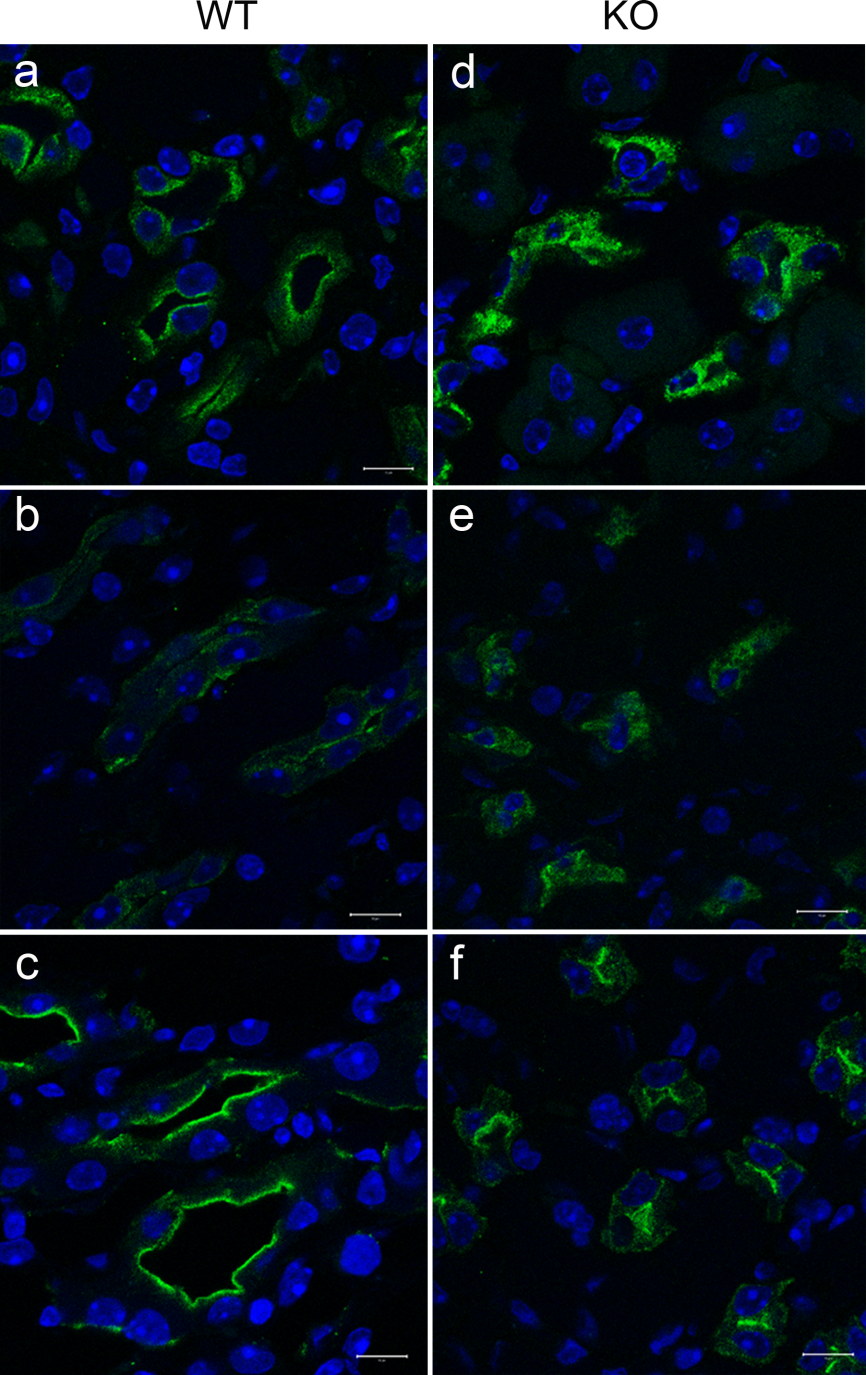
AQP2 acute response to vasopressin differed in slices of inner medulla from wild type and *Fxyd1* knockout mice. Kidney slices from WT (a-c) and KO (d-f) male mice were fixed without treatment (a,d), incubated in HBSS to wash out endogenous VP (b,e), and then treated with dDAVP for 15 min (c,f). Slices were post-fixed with 2% PLP and sectioned. The distribution of AQP2 was monitored by immunofluorescence. The absence of FXYD1 reduced shuttling of AQP2 between intracellular vesicles and subapical spaces and apical membrane. This figure is representative of 3 independent experiments. Nuclei were labeled with Dapi. Bars, 10 μm.

To test the hypothesis that the trafficking of AQP2 was altered in *Fxyd1*^-/-^ mice, we performed the same kind of time course experiments, but this was done in kidney slices from WT and *Fxyd1*^-/-^ mice for better time resolution, and for better experimental control of vasopressin addition and washout. Slices were suspended in HBSS (Hank’s balanced salt solution, glucose-containing physiological salts) equilibrated with a mixture of CO_2_ and O_2_. All except the baseline slices were incubated first at 37°C for 15 min in HBSS to allow washout of endogenous vasopressin. After equilibration, some of the slices were distributed to fresh vials and exposed to dDAVP for 15 min, followed by transfer to fresh vials with HBSS for timed washout and recovery. Upon completion of each step, slices were fixed with PLP followed by sectioning and immunostaining for AQP2. Disruption of AQP2 trafficking in *Fxyd1*^-/-^ mice can be seen in Fig 5. The differences in AQP2 localization in kidney from WT (a-c) and *Fxyd1*^-/-^ mice (d-f) were obvious at every time point. At baseline, in WT slices there was a mixture of intracellular and juxta-apical AQP2 (a), while in *Fxyd1*^*-/-*^ slices AQP2 was more uniformly intracellular (d). A similar but less pronounced pattern was observed after 15 min of washout of residual endogenous vasopressin (b and e for WT and *Fxyd1*^-/-^, respectively). Stimulation with dDAVP resulted in the majority of AQP2 accumulating at the apical membrane in both WT and *Fxyd1*^-/-^ mice, although the trafficking appeared less complete in the knockout (compare c and f).

**Fig 6:**
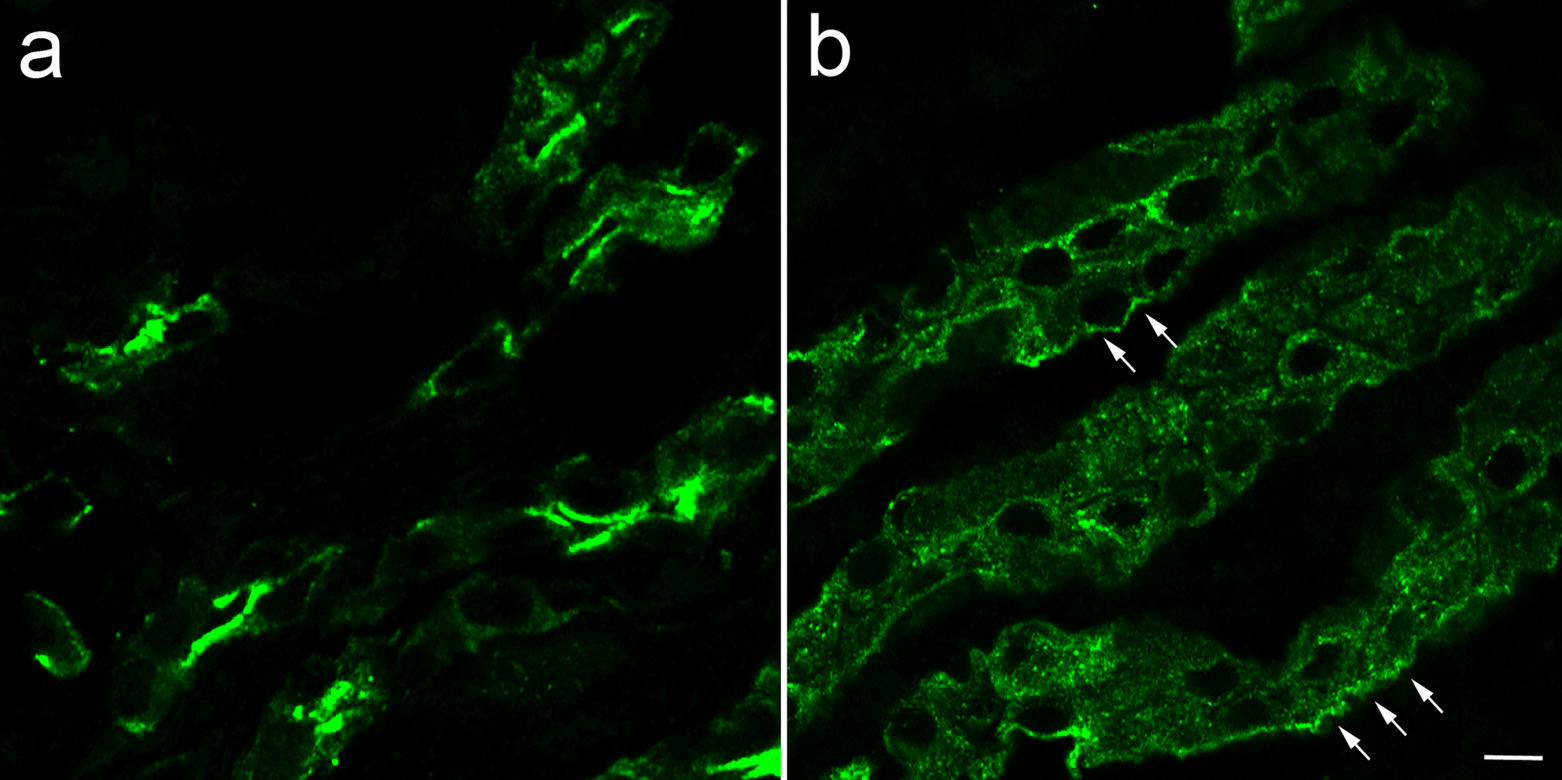
Washout of dDAVP revealed rapid redistribution of AQP2 in slices of inner medulla from *Fxyd1* knockout mice. Slices from WT (a) and KO mice (b) treated with dDAVP for 15 min as in Fig 5 were washed in HBSS for 60 min. Slices were fixed with PLP, sectioned, and stained for AQP2. The absence of FXYD1 resulted in less juxta-apical and more heterogeneous localization of AQP2 in kidney from KO mice (b). Arrows point to regions with AQP2 in proximity to the basolateral membrane. The images were enhanced and sharpened by subjecting the entire field to the high-pass filter in Adobe Photoshop. Bar, 10 μm.

Fig 6 shows that the absence of FXYD1 also affected AQP2 trafficking during recovery from experimental vasopressin treatment in slices. Tissue is shown 60 min after washout of vasopressin. In WT (a), washout resulted in a partial translocation of AQP2 from apical membrane into an intracellular compartment, but much was still concentrated at the apical membrane. In contrast, for *Fxyd1*^*-/-*^ slices (b), washout of dDAVP led to a more rapid reduction of AQP2 at the apical pole, and distribution to heterogeneous locations including close to the basolateral pole of the cells (arrows). The basolateral appearance differed from the starting position of AQP2 in the knockout (Fig 5D) and may reflect the prolongation of a transient basolateral step in the AQP2 trafficking cycle reported earlier [47–49]. Thus, the absence of FXYD1 did not completely prevent the upswing of AQP2 recruitment, but seemed to accelerate its reversal. The data imply that loss of FXYD1 interferes with the proper trafficking of AQP2, potentially explaining the impaired urine concentration observed in *Fxyd1*^*-/-*^ mice.

**Fig 7:**
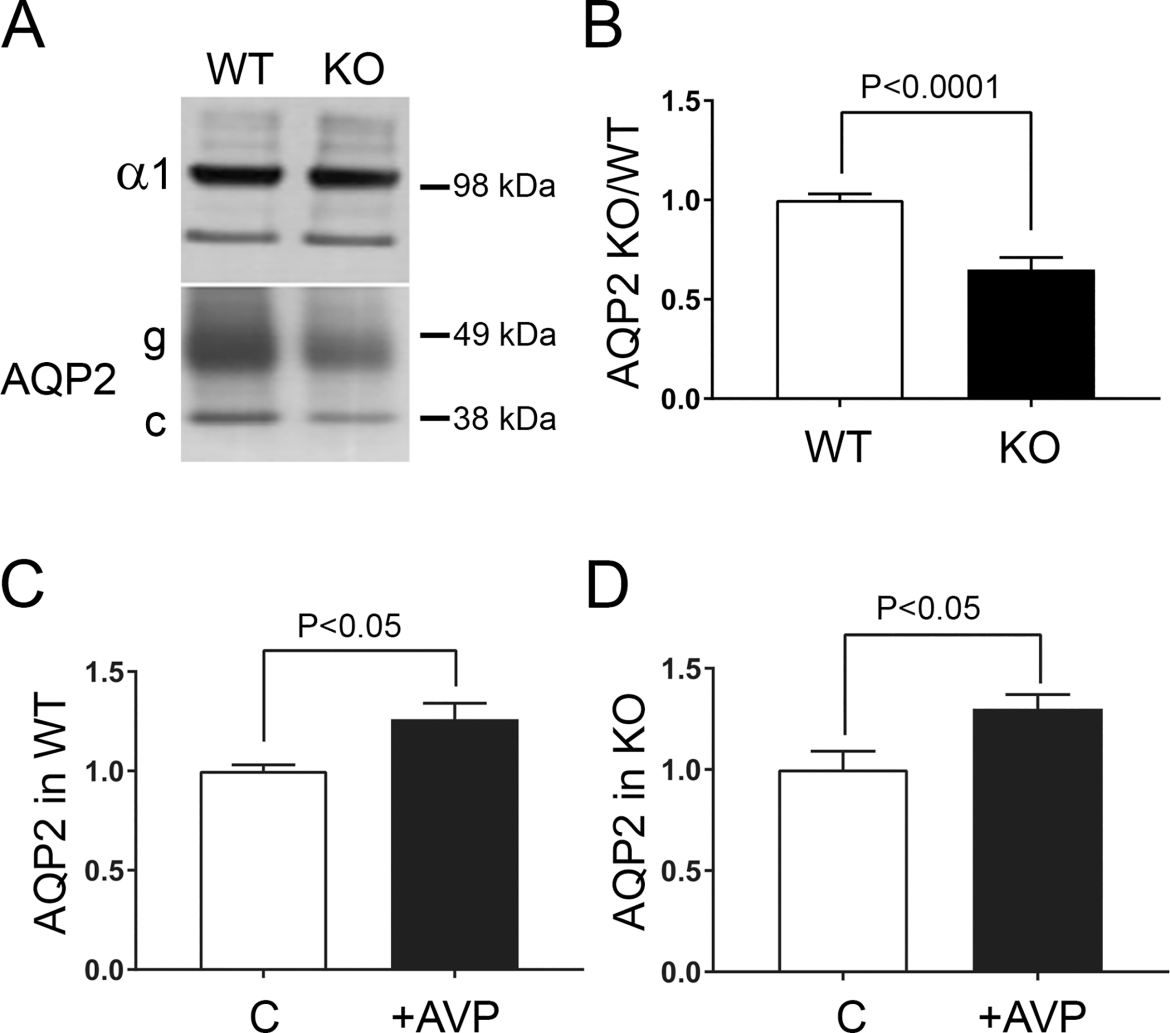
AQP2 abundance was reduced in FXYD1 knockout mice. (A, B) *Fxyd1*^-/-^ mice had a lower abundance of AQP2 in inner medulla. Inner medulla from WT and KO mice was obtained as lysates and tested on blots with specific antibodies [representative blot in (A)]. Both core (c) and glycosylated (g) species of AQP2 were reduced in KO animals. (B) quantification of results of three experiments. (C, D) Blot quantification of the relative changes in inner medullary AQP2 levels after 2 hour treatment of mice with dDAVP, 3 experiments. Stimulation with dDAVP resulted in a similar increase of AQP2 recovered in pelleted crude membranes in both WT (C) and KO (D). Bars are ± S.E.M. and significance was evaluated by Student’s *t*-test.

Another basic hypothesis to test was that a reduction of the amount of AQP2 in membranes might also contribute to the impaired urine concentration observed in the *Fxyd1*^-/-^ mice. Fig 7A shows representative western blots indicating that the abundance of AQP2 (both glycosylated and core forms of the protein) was lower in lysates and crude membrane fractions from the untreated inner medulla of *Fxyd1*^*-/-*^ animals compared to WTs. The mechanism for this is unknown, but the regulation of AQP2 transcription is very complex and depends on the physiological state (reviewed in [50]). Gel loading was normalized to the amount of the α1 subunit of Na,K-ATPase. The blots from three independent experiments were scanned and the data were expressed as the ratio of AQP2 relative to controls (Fig 7B). Injection of dDAVP caused a small increase in accumulation of AQP2 in crude plasma membrane fractions collected from both WT and *Fxyd1*^*-/-*^ mice 2 hours post-injection (Fig 7C and 7D). Whether this was due to new biosynthesis or to a shift of AQP2 into membranes pelleted under our conditions was not explored further. These data on AQP2 levels are not inconsistent with the marginally-complete vasopressin response to water deprivation seen in the knockout in Fig 1.

### Density differences of AQP2-containing membrane subfractions

After the observation of altered trafficking of AQP2, a key hypothesis was that there might be a detectable difference in the properties of AQP2-containing membrane subfractions. Vasopressin-stimulated accumulation of AQP2 in the apical membrane of IMCD cells is a transient event comprising regulated exocytotic fusion of subapical AQP2-bearing vesicles and reduced endocytotic internalization of AQP2. Rapid assembly/disassembly of vesicles with plasma membrane occurs via specialized structures, “membrane rafts”, that are enriched in sphingolipids and sterols and serve as platforms for membrane signaling and trafficking for multiple proteins [51,52]. By large-scale quantitative LC-MS/MS analysis, about half of the AQP2 was identified in the detergent-resistant membrane (DRM) domains from rat kidney collecting ducts [13]. Here we looked at the abundance of AQP2 in DRM fractions from IMCD in WT and *Fxyd1*^*-/-*^ mice and tested whether vasopressin-stimulated recruitment of AQP2 into DRM differed in the WT and knockout.

**Fig 8:**
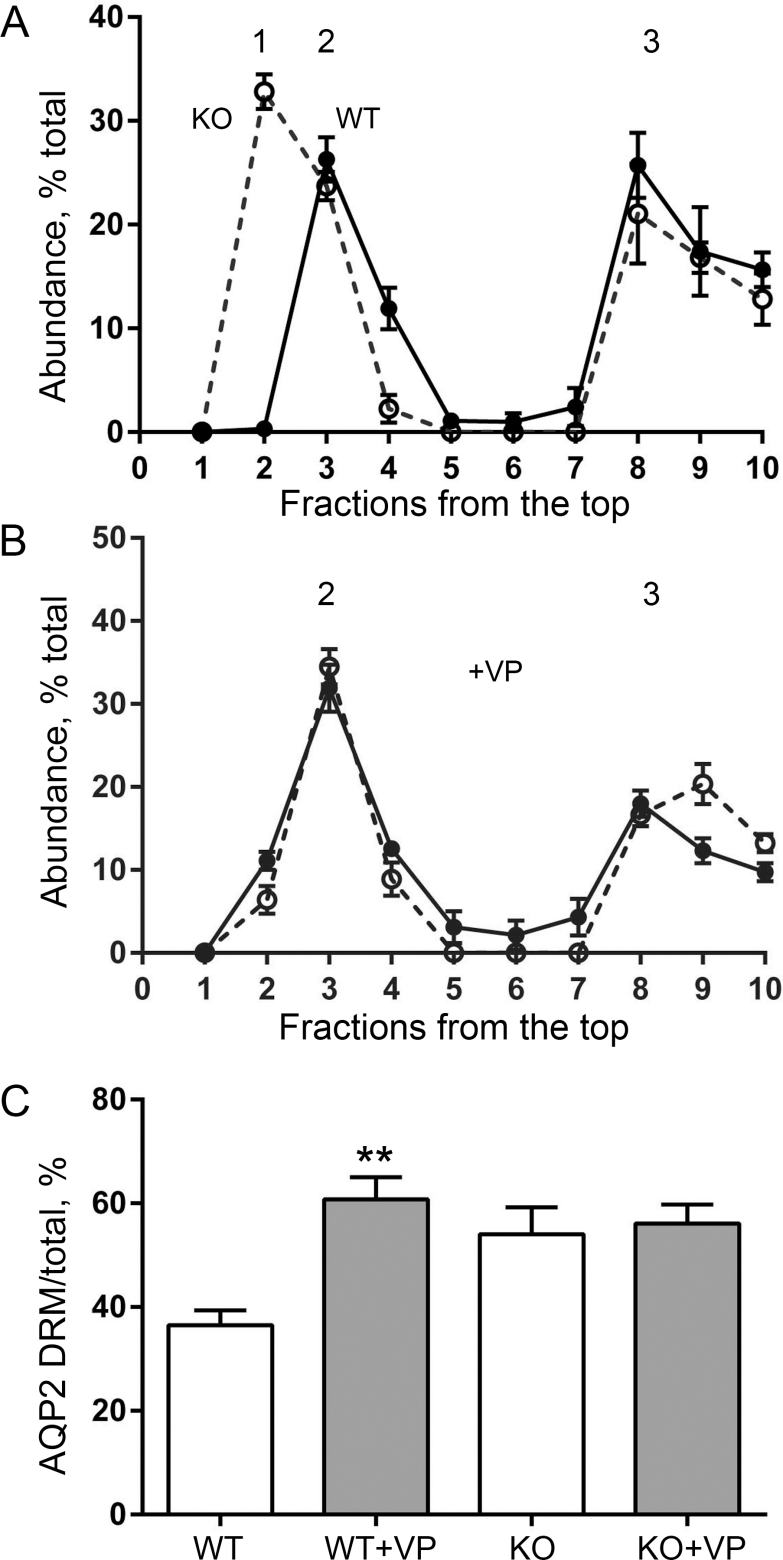
Flotation experiments revealed a difference in distribution of AQP2 in detergent-resistant membranes (DRM) from WT and knockout mouse. Membranes from inner medulla from wild type (solid lines in A and B) or FXYD1 KO male mice (dashed lines in A and B) treated with either vehicle (A) or dDAVP (B) for 1 hour were solubilized with 1% Triton, 30 min, 4°C, and separated on a step sucrose gradient of 5, 30, and 40% sucrose. DRM float near the top of the gradient (peaks 1 and 2). The distribution of AQP2 was quantified on Western blots with a cooled-CCD imager and expressed as % of total recovered AQP2 in each fraction. The data are mean ± SEM of four experiments. (A) Under basal conditions, the DRM AQP2 fraction from vehicle-treated knockout mice peaked in peak 1, while WT DRM appeared in peak 2. Roughly half of the AQP2 was in denser membranes (peak 3). (B) Stimulation with dDAVP caused redistribution of DRM AQP2 from the KO to peak 2. (C) Vasopressin-stimulated recruitment of AQP2 from peak 3 to DRM membranes in peak 2 occurred in WT but not in KO. (**) The difference is significant by Student’s *t*-test, P < 0.005, n = 4.

“Raft-like” domains have limited solubility in nonionic detergents such as Triton X-100 at low temperature, and proteins belonging to detergent-resistant fractions remain buoyant in sucrose density gradient centrifugation [52]. Fig 8 summarizes four replicate experiments in which membranes from inner medulla from wild type (solid line) or *Fxyd1*^-/-^ mice (dashed line) without (A) or with (B) injection of dDAVP (1 μg/ kg, 1 hour) were solubilized with 1% Triton X-100 at 4°C followed by separation by flotation in a sucrose step gradient. Consistent with the LC-MS/MS findings [13], AQP2 from WT mice was distributed approximately equally between two peaks characterized by different sucrose density. At high density (the initial position of the solubilized sample) was the so-called non-DRM membrane (peak 3), which also contained the α1-subunit of Na,K-ATPase (not shown). At low density was peak 2 where typical residents of DRM are usually found. Remarkably, the fractions containing AQP2-DRM from untreated *Fxyd1*^-/-^ mice were at even lower sucrose density (peak 1) than the DRM from WT animals, suggesting a major difference in the composition of the AQP2-containing fraction (Fig 8A). Stimulation with dDAVP, however, caused a shift of peak 1 from *Fxyd1*^*-/-*^ mice to the density of WT DRM in peak 2 (Fig 8B). In contrast, no change in the peak densities of either fraction was observed in WT mice regardless of stimulation with vasopressin. Quantitative analysis of AQP2 distribution along the gradients revealed another difference between genotypes, though. Stimulation with dDAVP in wild type mice apparently caused recruitment of AQP2 from peak 3 into DRM membranes in peak 2 (compare Fig 8A and 8B). No such recruitment was detected in gradients from the *Fxyd1*^-/-^ mice (Fig 8C). To phrase it another way, in WT mice, vasopressin’s greatest effect was to cause a shift from peak 3 to peak 2 (non-DRM to DRM), while for the knockout mice, the greatest effect was on a shift from peak 1 to peak 2 (DRM fractions of different density). The data indicate that the differences in trafficking of AQP2 in *Fxyd1*^*-/-*^ versus WT mice are accompanied by physical differences between the membrane compartments in which AQP2 resides. This is consistent with a role for FXYD1 in determining the specific trafficking pathway taken by AQP2.

### Sodium and water balance in *Fxyd1-/-* mice

Water intake and urine measurements in metabolic cages for 24 hours showed no polydipsia or polyuria, and no significant difference in urine osmolality (Table 1). This indicates that over 24 hours, the mice were in sodium and water balance. Circadian cycles in the kidney and other factors that may affect the physiology of the knockout are discussed below.

**Table 1:** The daytime defect in urine concentration in Fxyd1^-/-^ mice was compensated over 24 h.

24 hour urine collection	WT	KO	Statistical significance
**mOsm/30g**	3,117 ± 126, n = 18	3,442 ± 219, n = 18	P = 0.21
**volume, ml/30 g**	1.32 ± 0.13, n = 19	1.24 ± 0.12, n = 19	P = 0.67
**water intake, ml/30 g**	4.62 ± 0.33, n = 18	4.56 ± 0.29, n = 18	P = 0.89

Osmolality was measured in urine collected from male mice in metabolic cages and is expressed as mean ± SEM. N is the number of mice, and osmolality of each sample was measured in duplicate. In one experiment with females (five WT and five KO), urine osmolality was measured in five consecutive daily urine samples, and like the males, there was no significant difference between WT and KO (data not shown). Significance was evaluated by Student’s *t*-test.

## Discussion

### The cellular roles of FXYD1

The present work adds a new facet to our understanding of FXYD1’s subcellular distributions, which differ markedly depending on the cell. FXYD1, phospholemman, was discovered as a prominent target of protein kinases [16,53], and was only later shown to regulate Na,K-ATPase [21]. Unlike other FXYD proteins, FXYD1 has a record of alternative partners and of cytoplasmic locations, suggesting that it is a multi-faceted regulatory protein. In cardiac tissue, FXYD1 co-immunoprecipitated not only with Na,K-ATPase, but also with NCX1. When FXYD1 activated Na,K-ATPase, it inhibited NCX1, and it is likely that they are in a multimolecular complex in cardiac myocytes [29]. Furthermore, FXYD1 interacted with and stimulated a cardiac L-type calcium channel [30]. In the heart FXYD1 also dissociated from Na,K-ATPase and formed multimers in the membrane [31,54]. FXYD1 has been reported to be a caveolar-resident protein in heart, whether associated with Na,K-ATPase or not [55]. When expressed by transfection in MDCK cells, a polarized renal cell line, FXYD1 differed from all of the other FXYDs by being retained in the cytoplasm with an endoplasmic reticulum marker, and phosphorylation was required to mobilize it to the plasma membrane [56]. The terminal three-arginine motif of FXYD1, a known retention and trafficking motif, has been shown to affect its subcellular distribution [56,57]. Here in response to vasopressin, FXYD1 was conspicuously trafficked to the apical side of IMCD cells, away from the Na,K-ATPase. This is unprecedented: while FXYD1 is apical in choroid plexus (the epithelium that secretes cerebrospinal fluid), so is the Na,K-ATPase that is associated with it [39].

FXYD1 is phosphorylated by various kinases at a minimum of three clustered serine and threonine residues [18] and can be dephosphorylated by either PP1 or PP2A [19,20]. Phosphorylation of different residues can have similar consequences [23]. The vasopressin-induced trafficking of FXYD1 in IMCD was accompanied by FXYD1 dephosphorylation at the time points studied, as detected by antibodies recognizing phosphorylation at Ser63, and Ser68 and Ser/Thr69. In IMCD cells, the evidence presented here indicates that FXYD1 appears to be the target of a coordinated kinase/phosphatase system that is responsive to vasopressin. Further research will be needed to dissect the mechanism, because vasopressin binding to the V2R is now known to initiate a very complex response that includes not just activation of PKA, but also activation of protein phosphatases [58–60] and of cAMP-independent pathways ([61,62], reviewed in [63,64]).

### A trafficking role for FXYD1

Some prior reports implicate FXYD1 in physiological and pathological trafficking events [55,56]. In adipocytes, blockade of insulin-induced phosphorylation of FXYD1 with an alanine-substituted fragment of FXYD1 was accompanied by a reduction of GLUT4 (glucose transporter) recruitment to the plasma membrane [65]. More recently, anoxia/reoxygenation induced massive endocytosis of membrane in human stem cell-derived cardiac myocytes in a process that was highly dependent on palmitoylation of membrane proteins, including FXYD1. Endocytosis (estimated by membrane capacitance reduction) was reduced more than 50% in muscle strips in hearts from *Fxyd*^*-/-*^ compared to WT mice [66]. The mechanisms in the above cases, however, are not known. Proteins that associate with FXYD1 during its trafficking in IMCD here are also not known. Direct interaction of FXYD1 and AQP2, however, does not seem likely. 1) Co-immunoprecipitation of these two proteins was attempted, but was not detected (data not shown). 2) Double-label immunofluorescence did not show a robust colocalization of the two proteins except at the apical membrane. 3) FXYD1 was not detected by proteomic analysis of an immuno-isolated pool of AQP2-containing vesicles from rat IMCD, although FXYD2 and FXYD4 (presumptive cargo) were detected [67]. However, these are all negative results, and the possibility of direct interactions between FXYD1 and AQP2, possibly transient or mediated by a third protein, merits further investigation.

An intriguing possibility is that FXYD1 actively participates in trafficking because of its ability to be palmitoylated at two cysteines close to the membrane surface where the palmitoyl groups can insert [24,66]. Palmitoylation is strongly implicated in the targeting of both transmembrane and peripheral proteins to rafts [68,69], and rafts are in turn implicated in many steps of the intracellular trafficking of membrane compartments, including endosomes and caveolae as well as the trans-Golgi network [70].

### Aberrant trafficking of AQP2

The separation of detergent-resistant membranes by density gradient centrifugation provided physical evidence that AQP2 in the *Fxyd1*^-/-^ mouse was in different DRM fractions before and after vasopressin stimulation. At baseline it was in DRM peak 1, but after vasopressin injection it shifted to DRM peak 2. AQP2-containing DRM from WT were in peak 2 in both basal and vasopressin-stimulated states. This is consistent with the possibility that dephosphorylation of FXYD1 in response to vasopressin facilitates the distribution of AQP2 into a fraction such as apical recycling endosomes that are continually fusing with the apical membrane. Our immunofluorescence data suggest that dephosphorylated FXYD1 is recruited with AQP2 to the apical plasma membrane, and it is possible that there is an interaction between FXYD1 and AQP2 only in the apical membrane. WT AQP2-containing DRM from recycling endosomes and DRM from the apical membrane appeared to have the same flotation properties in our fractionation conditions, but *Fxyd1*^-/-^ DRM from endosomes were less dense, which could be explained if they lacked certain associated proteins. There are a number of plausible protein candidates associated with IMCD AQP2 trafficking endosomes ([13,67,71], reviewed in [64]).

In response to vasopressin injection or its addition to slices, the *Fxyd1*^-/-^ mouse could accumulate AQP2 in the IMCD apical membrane, although the efficiency was not as great as in WT. The differences in AQP2 distribution seen during washout of vasopressin were more striking. In knockout mice, there was apparently much more rapid routing (internalization) of a large fraction of AQP2 away from the apical plasma membrane to cytoplasmic locations and even to the basolateral pole of principal cells. In WT mice, this process was slower and substantial amounts of AQP2 were still detectable at the apical pole of these cells 1 hour after dDAVP washout. The defect thus may be in either arm of the trafficking cycle.

### Renal phenotype in the *Fxyd1* knockout

The dilute urine phenotype of the *Fxyd1* knockout mouse was detected in daytime urine samples, but the mice were in water and sodium balance over a 24 hour period and had normal blood pressure. There are several factors that may contribute to this. One of the most prominent is that rodents have a strong circadian rhythm whereby much more excretion occurs during the active phase (night) and little in the sleep phase (day) [72]. Mice were normally sacrificed between 6:00 and 8:00 ZT (time after lights-on), which is the lowest period of excretion of water and salt in rodents [72,73]. It is an attractive speculation that FXYD1 and its dephosphorylation may play a role in the reduction of renal output in the sleep phase by facilitating the subcellular trafficking of AQP2 in IMCD, downstream of circadian control. Interestingly, mice with knockout of the circadian gene *Clock* had dilute urine at ZT12 but not ZT2 [74]. The ability of the *Fxyd1* knockout to compensate urine output and osmolality over 24 hours, and its ability to concentrate urine during water deprivation, indicate that FXYD1 is, not unexpectedly, only one of many factors controlling AQP2 trafficking.

One might expect dilute daytime urine to be compensated by elevation of the concentration of nighttime urine, but that would be difficult to detect given the large difference between day and night urine production. Using measured diurnal urine volumes reported by Firsov [75,76] and our measurements in Fig 1 and Table 1, we estimated that the daytime fraction of the total osmolality burden excreted would be decreased from 10% to 6% in the *Fxyd1* knockout. To compensate for this, the nighttime osmolality burden would have to increase only from 90% to 94%.

The suppression of urination in daytime entails factors that could impact the physiological outcome for the *Fxyd*^*-/-*^ mouse. Daytime suppression of urine formation includes reduction of the glomerular filtration rate; reduction in aldosterone level; and phased fluctuations of many proteins, including V2R and aquaporins, with peaks during our sampling period of ZT6-8 [72–74]. There is even a low point in inner medullary osmolality at that time, which would reduce the driving force for water reabsorption and urine concentration, making the detection of this phenotype more likely in daytime [77]. The observed reduction of AQP2 levels in *Fxyd1* knockout compared to WT could also make a contribution that could be more important in daytime when interstitial osmolarity is lower than at night.

In summary, FXYD1 is universally believed to play the role of a signaling target, and like many such factors, it participates in different physiological processes. We suggest that its regulation of trafficking through a kinase/phosphatase pathway contributes to the fine-tuning of AQP2 accumulation in the apical membrane in IMCD, apparently by enhancing its retention. FXYD1, rather than being exclusively associated with Na,K-ATPase, was partly located in a phosphorylated state in a cytoplasmic compartment in IMCD of unstimulated mice. The total abundance of AQP2 was lower in the inner medulla in the *Fxyd1* knockout than in WT, but AQP2 showed a normal pattern of upregulation in response to injected vasopressin. Together, a lower expression level and reduced AQP2 retention in the apical membrane may be sufficient to explain the basal renal phenotype of dilute daytime urine in the knockout mice.

## Supporting information

S1 File Text and figures. Validation of the phosphosensitivity of antibodies employed.
(PDF)
