# Supplementary material for: Impaired AQP2 trafficking in Fxyd1 knockout mice: A role for FXYD1 in regulated vesicular transport
Source: PLoS One. 2017 Nov 20;12(11):e0188006. doi: 10.1371/journal.pone.0188006 (PMC5695786; doi:10.1371/journal.pone.0188006)
Supplement: S1 File — Validation of the phosphosensitivity of antibodies employed. (PDF) [file pone.0188006.s001.pdf]

Supplementary Figure A

Phosphospecificity of the PLM-C1 antibody.

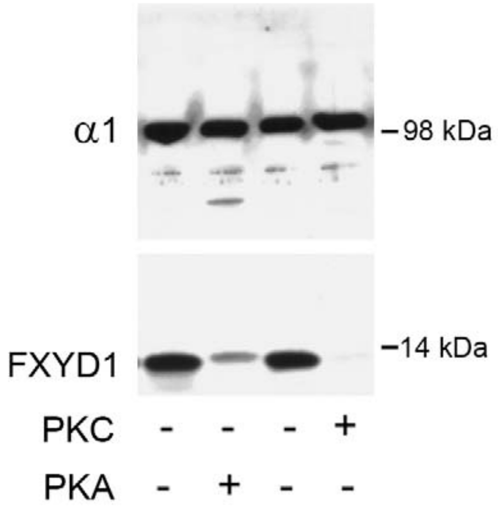

Phosphorylation with PKA reduced PLM-C1 stain by >95%, while phosphorylation with PKC abolished it.

Bovine choroid plexus, a very rich source of Na,K-ATPase α1 and FXYD1 (phospholemman) was homogenized and a crude microsome preparation was made by differential centrifugation. Tissue was homogenized in 250 mM sucrose, 30 mM imidazole, 1 mM EDTA pH 7.3. Pellets were collected for 15 min at 5,800 x g, rehomogenized, pelleted again, and the supernatants were combined and centrifuged for 1 h at 140,000 x g. The final pellets were resuspended in 320 mM sucrose, 20 mM Tris, 1 mM EDTA pH 7.2.

Samples were incubated at 1 μg protein in 10 μl of buffer containing 10 mM $MgCl_2$, 20 mM HEPES, 1 mM EGTA, pH 7.2, with 100 nM calcyculin A to block endogenous phosphatase activity, with and without exogenous protein kinases. Incubation was for 15-30 min at 30 ^oC. Protein kinase A catalytic subunit (Calbiochem, mouse recombinant) was used at 500-1000 units per sample. Protein kinase C (Calbiochem, rat brain) was used at 47.6 milliunits per sample, and the buffer additionally contained 0.5 mM $CaCl_2$, 80 μg phosphatidyl serine, 1 mM dithiothreitol, and 100 nM phorbol myristoyl acetate. Reactions were started by addition of 100 μM ATP, and stopped by addition of SDS-containing gel sample buffer.

The blot was divided, and the top stained with rabbit antibody anti-KETYY (gift of Dr. Jack Kyte, UCSD) at 1:5,000 as a loading control. The bottom was stained with rabbit antibody PLM-C1 at 1:5000.

Supplementary Figure B

Knockout mouse controls for C1 antibody specificity

A. Na,K-ATPase α antibody

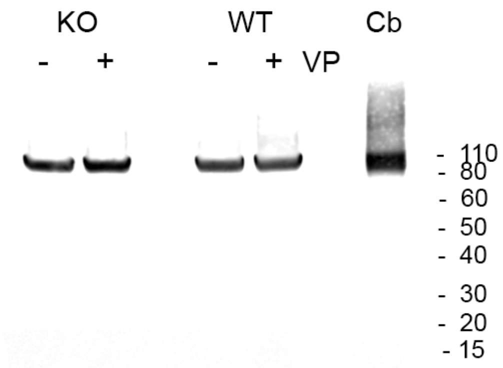

B. FXYD1 C1 antibody

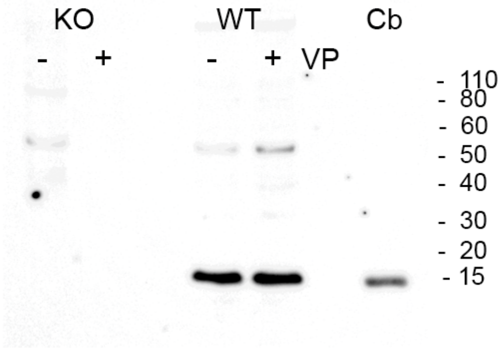

Wild type and knockout mice were treated with dDAVP in vivo for 2h as described. Panels A and B are full-length blots of IMCD membranes stained with either anti-Na,K-ATPase alpha-specific antibody 9A7, or with polyclonal anti-phospholemman antibody C1. Cb is membranes from mouse cerebellum used as a positive control.

In panel C, fixed sections of IMCD were stained with antibody C1 with anti-AQP2.

In all cases, stain for FXYD1 (phospholemman) was absent in the knockout.

C. Immunofluorescence

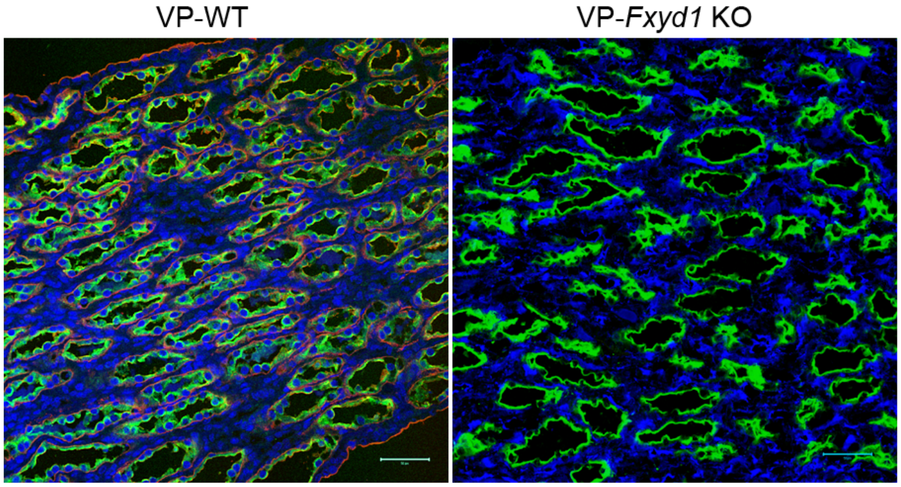

C1 antibody - red, AQP2 - green

Supplementary Figure C

Knockout mouse controls for C2 and phospho-specific antibodies.

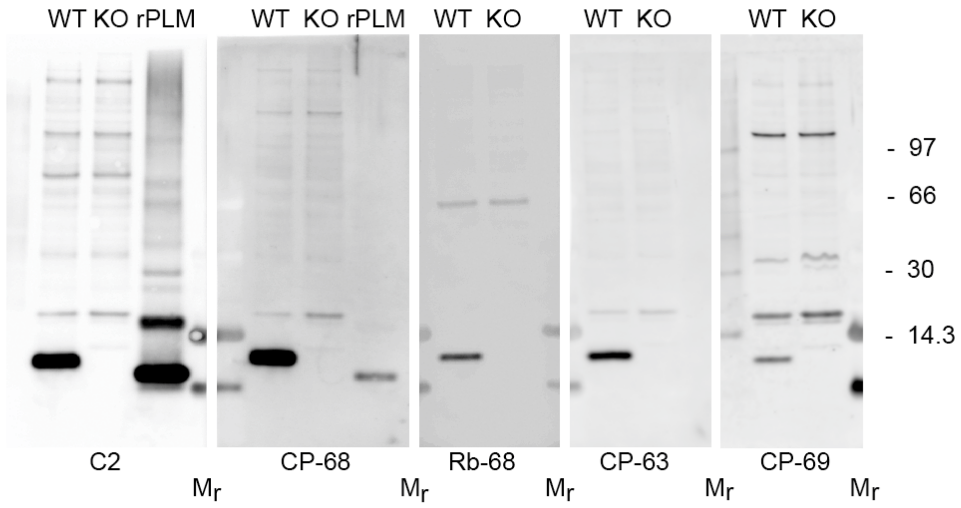

The tissue used in this experiment was mouse choroid plexus, a rich source of FXYD1. In some panels we also included recombinant mousse phospholemman (kind gift of Dr. Francesca Marassi, Sanford Burnham Prebys Medical Discovery Institute, La Jolla, CA), which ran slightly faster and also as a dimer. C2 and Rb-68 antibodies were raised in rabbits; the others were raised in sheep.
